# The role of early language abilities on math skills among Chinese children

**DOI:** 10.1371/journal.pone.0181074

**Published:** 2017-07-27

**Authors:** Juan Zhang, Xitao Fan, Sum Kwing Cheung, Yaxuan Meng, Zhihui Cai, Bi Ying Hu

**Affiliations:** 1 Faculty of Education, University of Macau, Macau, China; 2 School of Humanities and Social Science, The Chinese University of Hong Kong (Shenzhen), China; 3 School of Continuing Education, Hong Kong Baptist University, Hong Kong, China; 4 School of Psychology, Central China Normal University, Wuhan, China; Fordham University, UNITED STATES

## Abstract

**Background:**

The present study investigated the role of early language abilities in the development of math skills among Chinese K-3 students. About 2000 children in China, who were on average aged 6 years, were assessed for both informal math (e.g., basic number concepts such as counting objects) and formal math (calculations including addition and subtraction) skills, language abilities and nonverbal intelligence.

**Methodology:**

Correlation analysis showed that language abilities were more strongly associated with informal than formal math skills, and regression analyses revealed that children’s language abilities could uniquely predict both informal and formal math skills with age, gender, and nonverbal intelligence controlled. Mediation analyses demonstrated that the relationship between children’s language abilities and formal math skills was partially mediated by informal math skills.

**Results:**

The current findings indicate 1) Children’s language abilities are of strong predictive values for both informal and formal math skills; 2) Language abilities impacts formal math skills partially through the mediation of informal math skills.

## Introduction

Recently, increasing attention has been drawn to the development of early mathematical skills due to its importance on academic achievements and future occupational preparation. Based on previous studies, early mathematical skills are not only associated with later mathematical abilities, but also predictive of other academic aspects such as reading abilities [1, 2]. In addition, those who are functionally innumerate or suffering from dyscalculia, have more difficulties and obstacles in job hunting [3, 4]. As the poor performance in mathematics in early age can persistently hinder the academic development at school and influence the employment prospect in adulthood [3], it is important to understand the factors that may influence the development of mathematics from the early years.

### Mathematics and language

Among various academic skills, mathematics and language are the two most important domains [1]. Preschoolers’ early mathematical and language knowledge form the cornerstones for future learning, and provide motivational basis of later academic success [5, 6]. Although mathematics and language are two distinct subjects, relations between them have been studied extensively for years [1, 7, 8]. Previous studies revealed that mathematics and language were related to each other, after controlling for intelligence [8]. Language abilities not only predict children’s math skills in the long term, but also influence children’s early mathematical learning [7, 9].

#### The role of language components on mathematical learning

Language can be roughly divided into two categories, oral and literacy forms [10]. Oral language skills, including phonological, grammatical, and vocabulary abilities, are the prerequisite of the acquisition of literacy, which consists of reading and writing skills [11, 12]. Both of the two sub-domains of language are essential for developing mathematical skills [13, 14]. In what follows, the role of each language domain on mathematical learning is discussed.

For the influence of literacy on mathematics, previous studies mainly explored from the perspective of reading comprehension [7, 15]. Aiken noted that “it is generally recognized that not only do linguistic abilities affect performance in mathematics but that mathematics itself is a specialized language” (p.359) [16]. That is, learners need to construct the semiotic representations for mathematical concepts, symbols, and notions in order to connect new knowledge with previous mental representations [15]. However, mathematical language is different from our everyday language [17] and the difficulty and complexity of mathematical language also increase with grade levels. Especially for beginners of mathematics, the complexity of the verbal languages and semantic structure of word problems affect their understanding of the problems and strategies used to solve them because there is a lack of top-down processing [18]. Abedi and Lord found that children performed better in modified number word problems, of which the language was simplified, than in equivalent problems [7]. Similar result was found by Huang, Liu, and Chang that with the help of computer-assisted learning system, which explained the meaning and reminded the details of the word-based mathematical problems to students, low-achievers significantly improved their performance in solving these problems [19]. These findings indicate that literacy skills have influences on the learning and solving of word problems.

As for the impact of oral language on math learning, previous studies have shown a strong association between phonological processing and mathematical development. Phonological processing consists of three components: phonological memory, phonological retrieval, and phonological awareness. Each of them has been evidenced to be important for the development of mathematical skills [20]. According to the classical working memory model proposed by Baddeley, phonological memory involves the encoding and temporary storage of speech stimuli [21]. Encoding enables the conversion of arithmetic problems into verbal representations (e.g., encoding “2+3” to “two plus three”), while temporary maintenance prevents the verbal information from decaying so that it can be further processed (e.g., using procedural counting strategy to solve 2+3). Numerous studies thus far have found that children’s phonological memory span significantly correlates with their mathematical skills and predicts later mathematical performance [22–24].

Besides the encoding and storage of phonological memory, phonological processing also impacts mathematical abilities through phonological retrieval. The more efficient the child can access mathematically related phonological information (e.g., mathematical terms, operators, and answers) in long-term memory, the more able the child is to devote limited attention resources to completing necessary procedures during mathematical problem solving. Previous studies revealed that mathematically disabled children responded less accurately and more slowly when retrieving letter and simple mathematical computations compared with their normal counterparts [25].

Another aspect of phonological processing is phonological awareness, which refers to the ability to manipulate the sound structures of languages [26]. It has been a consensus in previous literature that phonological awareness is a significant correlate of individual differences in mathematical abilities [14, 20]. There are two possible reasons underlying the association between phonological awareness and mathematical abilities. First, phonological awareness may be linked to mathematical skills through number word learning [14]. Second, both phonological awareness and mathematical problem solving require substantial support of central executive control and phonological memory [20].

#### Informal and formal math

Similar to language, mathematical skills can also be conceptualized into two main components: informal math and formal math [27, 28]. Informal math refers to number sense and basic number concepts that are acquired by children before written mathematics is formally taught in school [27, 29]. It is manifested in their abilities to understand the magnitudes of sets of objects or of symbols, compare or estimate the magnitudes of small sets, associate the numerical value with the quantity of the set, etc. [30, 31]. Purpura and Ganley proposed that the development of informal math could be divided into three phases [28]. In the first phase, children learn to compare the magnitudes of two sets or objects and start to count sequentially. In the second phase, they learn to link numbers with corresponding quantities and number words. The third phase involves simple operations of number words (e.g., understanding that the sum of two whole-numbers is larger than either number). Different from informal math, formal math refers to skills or concepts that are taught in school and involves basic arithmetic such as addition and subtraction and more complicated calculations [27, 32, 33]. Informal math acts as the foundation of formal math because learning mathematics is an accumulative process, and the prior knowledge serves as the cornerstones of the new knowledge [34].

#### Mathematical learning in Chinese children

Although a substantial amount of research has been carried out to explore the relationship between language and mathematics, most research in this area focuses on children speaking alphabetic languages. As argued by LeFevre and Liu [35], different linguistic backgrounds may have an impact on the mathematical cognition. However, to the best of our knowledge, few studies have explored the predictive values of language factors on early math development in Chinese-speaking children. Studying Chinese-speaking children is important because Chinese language is different from alphabetic languages such as English in many aspects crucial for math learning. Chinese is semantically transparent and it makes the base ten structure of number names explicit [36]. In English, however, numbers are named in a much more implicit way. The difference is even more obvious for “teens” numbers [36]. For example, the Arabic number 13 is named as “十三” (ten-three) in Chinese, which can be easily obtained by simply adding 十 (meaning ten) to the number 三 (meaning three). However, in English, it is difficult to figure out how the number thirteen is derived from the number three. It was found that Chinese children learned “teens” numbers faster than their American counterparts [37]. That is, there may exist some differences in number words learning between Chinese children and their English-speaking counterparts. Furthermore, a previous study also found that Chinese children and adults outperformed their English-speaking counterparts in solving simple arithmetic problems [35]. The advantage of Chinese speakers in arithmetic calculations may be because that they are more likely to store the arithmetic facts (e.g., 2×3 = 6) in the auditory rote memory and use phonological codes to process the problems. Thus, the arithmetic facts tended to be retrieved from the phonological form by Chinese speakers and the arithmetic operation is more like a verbal-based task for them [38, 39]. Taken together, due to the discrepancies between Chinese and alphabetic languages, as well as the different strategies that Chinese speakers and alphabetic language speakers might use to solve arithmetic problems, it is worth exploring the role of language abilities on mathematical skills among Chinese-speaking population.

### The present study

As mentioned above, previous studies have found that language abilities play an important role in the acquisition of mathematical skills, and can predict later mathematical success. However, few, if any, studies have explored the impact of language abilities on informal and formal math skills respectively in Chinese children. The current study was conducted with two goals. First, it aimed to fill the abovementioned research gap by investigating whether Chinese-speaking children’s early language abilities, including both oral and literacy skills, were predictive of their informal and formal math skills respectively. Second, we aimed to explore the mechanism of how early language abilities would influence formal math skills. As informal math skills would serve as the foundation of formal math learning, we were especially interested in whether early language abilities would influence formal math skills directly, or through the mediation of informal math skills. We had two predictions as follows: 1), early language skills would be predictive of both informal and formal math skills in Chinese kindergartners. Specifically, language abilities might be more closely associated with informal math than with formal math skills given the semantic transparency of Chinese language and Chinese children’s reliance on phonological codes in arithmetic process; 2), the effect of Chinese-speaking children’s language abilities on early formal math skills would be partially mediated by their informal math skills. That is, informal math skills would act as a mediator between early language abilities and formal math skills.

## Method

### Participants

The participants were 2012 K-3 children (958 girls and 1054 boys, mean age = 6.65 years, SD = .14) recruited from 60 kindergartens in three cities in Guangdong province, China. As part of a more extensive research project, the three cities were selected because they represent different levels (above average, average, below average) of economic development of Guangdong Province. All the students were typically developing children and informed consent was obtained from their parents before the formal testing was performed. The experimental procedures were approved by the Ethics Committee of University of Macau. All methods were carried out in accordance with the approved guidelines and regulations. Each child was assessed for math skills, language abilities, and non-verbal intelligence.

### Measures

Seven tasks were adopted to assess children’s early math skills. The informal math score was obtained by adding the raw scores in the first five tasks, namely object counting, forward counting, backward counting, missing number, and numerical magnitude comparison. The formal math score was computed by summing up the raw scores in the remaining two tasks, namely addition, and subtraction. These tasks, together, could provide a comprehensive picture of children’s knowledge of counting (including counting principles and counting sequence), number knowledge (i.e., numerical magnitude of numbers), and number calculation (including addition and subtraction). Based on the “Guideline of the Learning and Development of Children Aged 3–6” (2012) published by the Ministry of Education of China, the items used in previous studies were modified, so that each task consisted of both average and challenging items for our participants. This allowed us to differentiate children who were under- and over-achieving in math from those who were average-achieving. There were more test items for the last four math tasks (i.e., missing number, numerical magnitude comparison, addition, and subtraction) than the first three tasks (i.e., object counting, forward counting, and backward counting) because the last four tasks assessed more advanced concepts and skills, necessitating more items to yield more variability in children’s scores. Different from some previous studies [40–42] in which experiments asked questions and children gave answers orally, children in the present study completed the math tasks in paper-and-pencil format, because they were competent and used to do so.

For the early language skills, listening comprehension and character dictation tasks were chosen. Listening comprehension is a comprehensive measure of oral language skills [40] and character dictation task is a typical measure of Chinese literacy skills [41]. As mentioned above, the two subdomains of language were both essential in developing mathematical skills and thus, were measured in the present study.

#### Object counting

This task was adopted from the study of Cheung and McBride-Chang [42]. In each item, children were instructed to count and write down the number of animals presented. There were three test items (11, 16 and 17 animals respectively), and the maximum score was 3.

#### Forward counting

This task was similar to the Counting From task of Chard and his colleagues [43]. In each trial, three numbers were presented in ascending order followed by four missing numbers (e.g., 16, 17, 18, __, __, __, __). Children were asked to write down what the following four numbers should be. There were three test items in total, and the targeted numbers ranged from 19 to 104. Each correctly answered number was scored as 1, and the maximum score was 12.

#### Backward counting

This task was modified from the Counting From task used by Chard and his colleagues [43] and the Counting Backward task used by Malofeeva and her colleagues [44]. It was similar to the forward counting task mentioned above, except that all numbers were presented in descending order (e.g., 65, 64, 63, __, __, __, __). Three test items were included, and the targeted numbers ranged from 14 to 96. The maximum possible score the child could get was 12.

#### Missing number

This task was similar to those used in previous studies [42, 45–47]. Three numbers were presented in ascending order and one of them was missing (e.g., __, 34, 35). The missing number could appear anywhere among the three numbers. Children’s task was to figure out what the missing number was. There were five test items, and the targeted numbers ranged from 33 to 332. The maximum score children could get was 5.

#### Numerical magnitude comparison

This task resembled those adopted in previous studies [42, 46, 47]. In each item, two numbers (e.g., 56 and 26) were presented and children were asked to judge which number represented greater quantities. Five test items (56/26, 24/42, 105/150, 363/636, 484/448) were presented in total and the maximum score was 5.

#### Addition

This task was like the addition/subtraction combinations task of Bryant and her colleagues [45]. Children were asked to do some simple arithmetical additions of two whole numbers (e.g., 2 + 3) and write down the answers. There were five test items, with answers ranging from 5 to 37. The maximum possible score was 5.

#### Subtraction

This task was similar to the addition/subtraction combinations task of Bryant and her colleagues [45]. Children were asked to do some simple arithmetical subtractions of whole numbers (e.g., 10–2) and write down the answers. Similar to addition, five test items were presented, with answers ranging from 2 to 31. The maximum score was 5.

Language abilities were assessed in both receptive and productive ways through listening comprehension and Chinese character dictation task, respectively.

#### Listening comprehension

**Fig 1:**
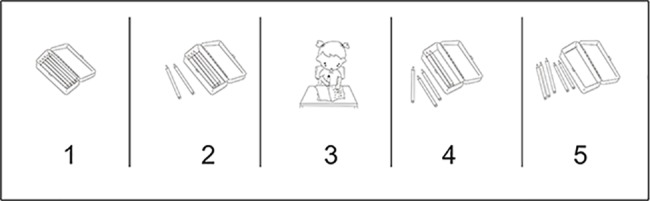
Example for listening comprehension task.

In each trial, a simple sentence was aurally presented and children were asked to pick up one from five pictures that best depicted the sentence as shown in Fig 1. A similar task was used in previous studies to assess Hong Kong Chinese children’s language comprehension [48]. There were 25 items and the maximum score was 25.

#### Chinese character dictation

In each trial of the task, children were asked to write down certain character in a multi-character word and there were 15 characters in total. Children would get a score of 2 if they wrote the character correctly and get a score of 1 if the character was partially correct. Otherwise, the score would be 0. The possible maximum score was 30 in this task.

#### Nonverbal intelligence

Raven’s Progressive Matrix (Set A and Set B) was used to tap children’s nonverbal intelligence [49]. Two sets of tests were administered and each set contained 12 items. In each trial, children were asked to choose one from five figures that best fit a visual geometric design with a missing piece. Children would get a score of 1 if their choice was correct and the maximum total score was 24.

### Procedure

Children completed all the tasks in the kindergarten classrooms under the instructions of the experimenters who were graduate students pursuing master degree in early childhood education. The whole testing was divided into three testing sessions, with each lasting for about 30 minutes.

## Results

### Preliminary analyses

Table 1 shows the means and standard deviations for all tasks as well as the overall informal, formal, and language scores. In addition, the zero-order correlations among the variables and their partial correlations when controlling for nonverbal intelligence were shown in the left bottom and right top of the table, respectively. As shown in the left bottom of Table 1, the overall language abilities were significantly and strongly associated with both informal and formal math skills (*r* =. 74 and .67 for informal and formal math, respectively). Further statistical analysis found a significant difference between the two correlations (*z* = 4.43, *p*<0.001), suggesting that language abilities were more closely related to informal than formal math skills. Even with nonverbal intelligence statistically controlled, the overall language abilities were still significantly correlated with informal and formal math scores (*r* =. 64 and .57 for informal and formal math, respectively, as shown in the right upper of Table 1). Moreover, the two partial correlation coefficients were significantly different (*z* = 3.51, *p*<0.001).

**Table 1:** Descriptive statistics, correlations, and partial correlations among all tasks.

	1	2	3	4	5	6	7	8	9	10	11	12	13
1. Object counting	-	.32	.16	.25	.29	.29	.23	.39	.29	.19	.33	.33	
2. Forward counting	.39	-	.42	.59	.57	.50	.45	.84	.53	.37	.49	.54	
3. Magnitude comparison	.25	.53	-	.43	.46	.44	.38	.62	.45	.43	.33	.45	
4. Backward counting	.34	.67	.54	-	.59	.52	.51	.88	.57	.40	.43	.51	
5. Missing number	.38	.66	.56	.68	-	.55	.52	.76	60	.37	.48	.53	
6. Addition	.37	.60	.54	.62	.64	-	.62	.62	.89	.38	.46	.52	
7. Subtraction	.31	.55	.49	.60	.62	.69	-	.58	.91	.35	.46	.51	
8. Informal math skills (1–5)	.46	.88	.70	.91	.82	.71	.67	-	.67	.48	.55	.64	
9. Formal math skills (6–7)	.37	.63	.56	.67	.69	.91	.92	.75	-	.40	.51	.57	
10. Listening comprehension	.30	.52	.56	.55	.53	.53	.50	.63	.56	-	.31	.71	
11. Dictation	.39	.58	.44	.53	.57	.55	.55	.63	.60	.46	-	.89	
12. Language abilities (10–11)	.41	.64	.57	.63	.64	.62	.61	.74	.67	.79	.89	-	
13. Nonverbal intelligence	.26	.43	.42	.46	.47	.43	.41	.53	.46	.56	.38	.53	-
Mean	2.58	9.05	3.47	7.45	3.54	3.23	2.73	26.09	5.95	13.58	20.27	33.60	13.50
STD	.71	3.62	1.68	4.60	1.65	1.59	1.69	10.22	3.01	5.52	7.71	11.48	5.02

Note. N = 2012. All the correlations were significant at *p* < .001. The left bottom shows the zero-order correlations among the variables, and the right top shows their partial correlations when controlling for nonverbal intelligence.

### Regression analyses

To test the unique contribution of overall language abilities to informal and formal math skills, hierarchical regression analyses were conducted, with the overall informal and formal math scores as dependent variables. Since language abilities and math skills might be associated with each other due to some general cognitive or demographic factors, gender, age, and nonverbal intelligence were entered in Step 1 as control variables. The overall language score was added to the regression model in Step 2. Table 2 shows that the overall language abilities significantly predicted 31% unique variance of informal math (*β* = 0.66, *t* = 36.25, *p* < .001) when gender, age and nonverbal intelligence were statistically controlled, with the whole model accounting for 58% of the total variance. For formal math, as shown in Table 2, the overall language abilities explained 26% unique variance (*β* = 0.61, *t* = 29.78, *p* < .001) with the same variables controlled, and the whole model accounted for 47% of variance.

**Table 2:** Two-Step hierarchical regression models predicting informal math and formal math skills from language abilities.

Outcome	Step	Variables	β	t	R^2^	ΔR^2^
Informal Math	1	Age	-.01	-.79	.27	.27*
		Gender	.07	4.67*		
		Non-verbal IQ	.17	9.55*		
	2	Language abilities	.66	36.25*	.58	.31*
Formal Math	1	Age	-.01	-.67	.21	.21*
		Gender	.06	-3.55*		
		Non-verbal IQ	.14	6.75*		
	2	Language abilities	.61	29.78*	.47	.26*

Note.

*p < .001

### Multilevel mediation modeling analyses

**Fig 2:**
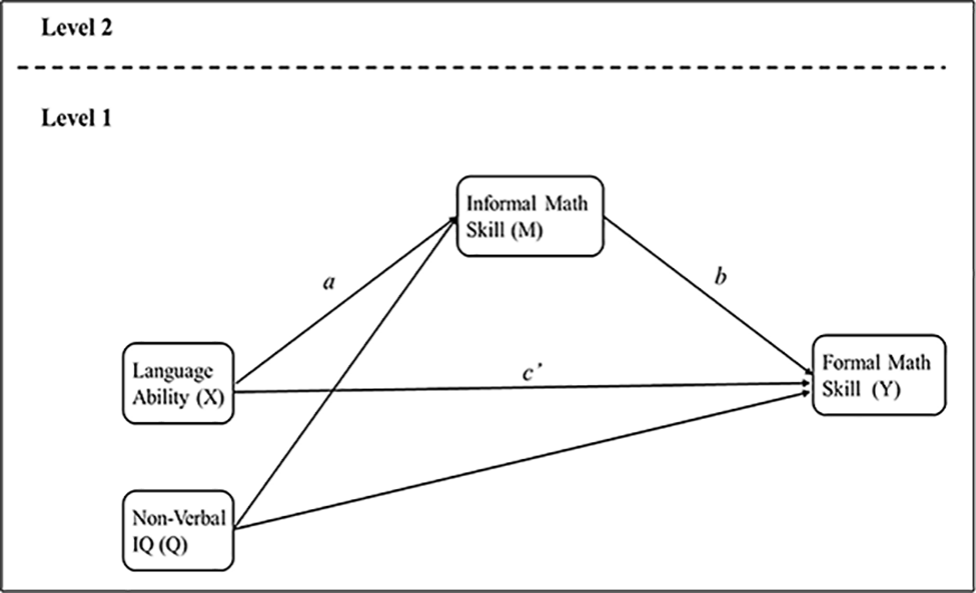
Proposed lower level mediation model of language ability, informal math skill and formal math skill.

To further explore the relationship between language and mathematics, a model in which children’s informal math skills mediate the relationship between children’s language abilities and formal math skills was constructed. In the mediation analysis, to tease apart the possible influence of the general cognitive abilities on both informal math and formal math skills, we included nonverbal intelligence as the control variable in the mediation analysis model. As participants were from the same schools, the data were nested. Multilevel modeling analysis was therefore conducted to account for possible similarities among the participants from the same school [50] (see Fig 2). The hypothesized model was estimated with separate regression equations based on the multilevel mediational analysis procedures described by Krull and MacKinnon [51].

These regression equations were estimated by using HLM6, a statistical analysis software designed for multilevel analysis. Missing values (less than 2%) were imputed using the approach of series mean in SPSS. All variables in the study were standardized before conducting the hierarchical linear modeling analyses. The predictors in the models were group-centered at level 1. The analytic procedures were as follows: first, school clustering effect was assessed for the three variables (language ability, informal math skill, and formal math skill). Findings of statistically significant between-school variability would support continued multilevel modeling analysis. Second, test of the mediation model at the individual level in which children’s informal math skills mediate the relationship between children’s language abilities and formal math skills. The testing process as described in Wen and Ye [52] was used to estimate the mediation effect, their standard errors and their level of statistical significance.

Table 3 shows the means, maximum, minimum, reliability (Cronbach coefficient αs), standard deviations, and zero-order correlations of the variables, and the estimates of the intraclass correlations (ICC) for each variable. The ICC estimates the proportion of the total variance in the variable that is between schools. It was found that the estimated ICCs were all very large, indicating that the between-school variability was substantial, and it was necessary to conduct multilevel mediation modeling analyses for the data.

**Table 3:** Descriptive statistics, zero-order correlations and intraclass correlations (ICC) of the variables.

	M	SD	Min	Max	Reliability	1	2	ICC
Student level (n = 2012)								
1. Language abilities	33.60	11.48	0	60.50	0.903			0.50
2. Informal math skills	26.09	10.22	0	37.00	0.864	.74**		0.43
3. Formal math skills	5.95	3.01	0	10.00	0.864	.67**	.75**	0.44
School level (n = 60)								

Note.

**p < .01

The findings in Table 4 provided evidence for a positive and significant relationship between children’s language abilities and formal math skills (c = 0.427, SE = 0.024, *p*<0.001), indicating that children’s language abilities can positively influence their formal math skills. Then we proceeded to estimate the mediation effect. The coefficients *a* (*a* = 0.540, SE = 0.022, *p*<0.001) and *b* (*b* = 0.498, SE = 0.022, *p*<0.001) were both significant, indicating that children’s informal math skills have a mediation effect (ab, product of a and b) on the formal math skills. That is, children’s language abilities influenced their informal math skills, which in turn influenced their formal math skills. The coefficient *c'* (*c'* = 0.158, SE = 0.024, *p*<0.001) remained statistically significant once the mediation effect was considered, suggesting that there still existed a direct effect from language abilities to formal math skills beyond the mediation effect. Taken together, the results were consistent with the hypothesis that the effect of language abilities on formal math skills was partially mediated by informal math skills, and the mediating effect accounted for 62.98% of the total effect.

**Table 4:** Results from multilevel mediation modeling analysis.

parameter	Model 1			Model 2			Model 3		
	*Y*_*ij*_ = *B*_0*j*_ + *cX*_*ij*_ + *β*_2*j*_*Q*_*ij*_ + *r*_*ij*_			*M*_*ij*_ = *B*_0*j*_ + *aX*_*ij*_ + *β*_2*j*_*Q*_*ij*_ + *r*_*ij*_			*Y*_*ij*_ = *B*_0*j*_ + *c*^′^*X*_*ij*_ + *bM*_*ij*_ + *β*_2*j*_*Q*_*ij*_ + *r*_*ij*_		
	Estimate	SE	t	Estimate	SE	t	Estimate	SE	t
*B*_0*j*_	0.044	0.088	0.5	0.028	0.086	0.3	0.044	0.088	0.5
*β*_2*j*_	0.119	0.020	6.0*	0.132	0.019	7.1*	0.053	0.018	3.0*
c	0.427	0.024	17.8*						
a				0.540	0.022	24.3*			
b							0.498	0.022	33.0*
c´							0.158	0.024	6.5*

Note.

*p < .001

## Discussion

The study generated two main findings. First, language abilities were important for developing math skills in Chinese-speaking children. Specifically, language abilities were able to significantly predict both informal and formal math skills. Furthermore, language abilities were linked differently to different math skills, being more closely associated with informal math than with formal math. Second, the association between language abilities and formal math skills was partially mediated by informal math skills. There could be several possible explanations for the first finding. First, given that math is a special kind of language [16], the process of acquiring informal math skills shares some level of similarity with that of language skills [53]. To build up vocabulary, young children connect words to objects, events and concepts. When they come to acquire number words, they have to understand that each number word represents a certain amount of quantities and with richer vocabulary, young children might practice more and performance better [53]. Second, young children’s general language abilities can support their understanding of the meaning of terminologies involved in informal math, such as “larger”, “smaller”, “more”, “less”, “equal to”, “ascending” and “descending” [53]. Thirdly, given that linguistic transparency of the Chinese numeration system may facilitate the learning of place value [54], young children with better Chinese language abilities might have better mastery of counting and number sequences.

Besides, there are at least two reasons for the greater correlation found between language and informal math as compared to language and formal math. One possible reason is that although language abilities and formal math skills involve the mapping between words and written symbols [33], the verbal coding procedure is not sufficient for arithmetic operations. The second reason is related to the cultural-specific mathematical teaching method in China. Chinese parents and teachers are inclined to emphasize the rote memorization of simple arithmetic facts and Chinese children are exposed to language-based memorization through the manipulation of real objects from an early age [38]. This perhaps explains why language abilities are more strongly associated with informal math than with formal math skills.

Meanwhile, results of mediation analyses suggested that language abilities also had indirect influence on formal math skills through informal math skills. Among past studies, informal math skills were often found to be precursors of the development of formal math skills [55, 56]. As proficiency in language skills can promote informal math skills, such as oral counting and understanding mathematical story problems, which are essential for performing formal arithmetic operations. Thus, the development of formal math skills can therefore benefit from language abilities via informal math skills. Findings of the present study have both theoretical and practical implications. First, the present study provides further empirical support for the existing models on the developmental precursors of math skills. The positive relation of children’s language to math skills not only exists among Western children speaking alphabetic languages, but also among Chinese young children who use a non-alphabetic language system. Moreover, like their Western peers, Chinese young children’s informal and formal math skills seem to follow distinct developmental pathways. Herein, the present study extends previous research in the area by suggesting that language skills have both direct and indirect effects on formal math skills. Second, in view of the partially mediating effect of informal math skills on the relation between Chinese language and formal math skill among Chinese young children, when providing remediation to students who struggle with formal math skills, the present findings suggest that improving language and informal math skills could improve formal math skills [57, 58], for the reason that math and language interventions for young children have been found to significantly enhanced their math knowledge and improve their later math performances [59, 60]. Finally, given that children often start to learn informal math skills spontaneously from their everyday experiences before entering school [33], our findings suggest that developing parent education programs on how to promote young children’s informal math skills through interactive parent-child activities might improve their formal math skills. As some parents often miss the opportunities to incorporate math contents into their discourse with children during informal home numeracy activities [42, 61], it is important to provide some trainings to them. For example, parents can be introduced some examples of math games that meet the developmental needs of young children of different ages, the types of concrete materials that help young children learn math concepts effectively, and some questions that can be asked to provoke young children to practice different math skills during home activities. Indeed, parent training of this kind has been found to be effective in promoting young children’s math skills among past studies [42, 62].

Nevertheless, the present study had limitations in its scope of investigation. For example, the present study only adopted a listening comprehension task and a Chinese character dictation task to assess children’s language skills. In future studies, the relationships of a wider variety of early language and literacy abilities with Chinese children’s informal and formal math skills should be explored. For example, given that the Chinese numeration system provides a relatively more transparent representation of numerical quantities than English, and the Chinese writing system is logographic rather than alphabetic, it would be meaningful to investigate the extent to which phonological processing abilities and print knowledge predict math skills in Chinese young children. Moreover, in the present study, children’s formal math skills were assessed by using number combination problems presented in written expressions. In fact, children’s understanding of addition and subtraction principles can also be measured with problems with manipulation of concrete objects and story problems [63]. It might therefore be interesting to investigate whether the predictors of addition and subtraction skills among Chinese young children vary when using different types of measures, so as to understand the extent to which the linguistic transparency of Chinese numbers benefits arithmetic operations. Finally, as discussed by [56], in addition to quantitative and linguistic precursors, some early math skills (such as numeration and calculation skills) can be predicted by spatial precursors. Given that visual skills are critical for early Chinese literacy acquisition [64], future researchers can compare the relative contribution of spatial skills, as compared to literacy skills, to the development of informal and formal math skills among Chinese young children.

## Conclusion

Though further work is required to obtain a more comprehensive understanding of the developmental precursors of math skills of children from different linguistic backgrounds, the present study has provided a good start for untangling the relations among language, informal math, and formal math skills among young children speaking Chinese, which is non-alphabetic and semantically transparent. As shown in the present study, language abilities seem to have a closer connection with informal than formal math skills. Moreover, language abilities have both direct and indirect influences on formal math skills. These findings are believed to provide important insights on ways to promote young children’s different types of math skills.

## Supporting information

S1 Table Relevant data underlying the findings described in manuscript. (XLSX)

## References

[1] Duncan GJ, Dowsett CJ, Claessens A, Magnuson K, Huston AC, Klebanov P, et al School readiness and later achievement. Developmental Psychology. 2007; 43: 1428 doi: 10.1037/0012-1649.43.6.1428 18020822 10.1037/0012-1649.43.6.1428

[2] Koedel C, Tyhurst E. Math skills and labor-market outcomes: Evidence from a resume-based field experiment. Economics of Education Review. 2012; 31: 131–140.

[3] Geary DC. Early foundations for mathematics learning and their relations to learning disabilities. Current Directions in Psychological Science. 2013; 22: 23–27. doi: 10.1177/0963721412469398 26229241 10.1177/0963721412469398 PMC4517838

[4] Shalev RS, Auerbach J, Manor O, Gross-Tsur. Developmental dyscalculia: prevalence and prognosis. European Child & Adolescent Psychiatry. 2000; 9: S58–S64. 10.1007/s007870070009 11138905

[5] Miller MR, Müller U, Giesbrecht GF, Carpendale JI, Kerns KA. The contribution of executive function and social understanding to preschoolers’ letter and math skills. Cognitive Development. 2013; 28: 331–349.

[6] Romano E, Babchishin L, Pagani LS, Kohen D. School readiness and later achievement: Replication and extension using a nationwide Canadian survey. Developmental Psychology. 2010; 46: 995 doi: 10.1037/a0018880 20822218 10.1037/a0018880

[7] Abedi J, Lord C. The language factor in mathematics tests. Applied Measurement in Education. 2001; 14: 219–234.

[8] De Smedt B, Taylor J, Archibald L, Ansari D. How is phonological processing related to individual differences in children’s arithmetic skills?. Developmental Science. 2010; 13: 508–520. doi: 10.1111/j.1467-7687.2009.00897.x 20443971 10.1111/j.1467-7687.2009.00897.x

[9] McClelland MM, Cameron CE, Connor CM, Farris CL, Jewkes AM, Morrison FJ. Links between behavioral regulation and preschoolers’ literacy, vocabulary, and math skills. Developmental Psychology. 2007; 43: 947 doi: 10.1037/0012-1649.43.4.947 17605527 10.1037/0012-1649.43.4.947

[10] Berninger VW. Development of language by hand and its connections with language by ear, mouth, and eye. Topics in Language Disorders. 2000; 20: 65–84.

[11] Muter V, Hulme C, Snowling MJ, Stevenson J. Phonemes, rimes, vocabulary, and grammatical skills as foundations of early reading development: evidence from a longitudinal study. Developmental Psychology. 2004; 40: 665–681. doi: 10.1037/0012-1649.40.5.665 15355157 10.1037/0012-1649.40.5.665

[12] Shanahan T, MacArthur CA, Graham S, Fitzgerald J. Relations among oral language, reading, and writing development In: Handbook of writing research. New York: Springer; 2006 pp. 171–183.

[13] Fletcher JM. Predicting math outcomes reading predictors and comorbidity. Journal of Learning Disabilities. 2005; 38: 308–312. doi: 10.1177/00222194050380040501 16122061 10.1177/00222194050380040501

[14] Krajewski K, Schneider W. Exploring the impact of phonological awareness, visual–spatial working memory, and preschool quantity–number competencies on mathematics achievement in elementary school: Findings from a 3-year longitudinal study. Journal of Experimental Child Psychology. 2009; 103: 516–531. doi: 10.1016/j.jecp.2009.03.009 19427646 10.1016/j.jecp.2009.03.009

[15] Duval R. A cognitive analysis of problems of comprehension in a learning of mathematics. Educational Studies in Mathematics. 2006; 61: 103–131.

[16] Aiken LR. Language factors in learning mathematics. Review of Educational Research. 1972; 42: 359–385.

[17] Schleppegrell MJ. The linguistic challenges of mathematics teaching and learning: A research review. Reading & Writing Quarterly. 2007; 23: 139–159.

[18] Ashkenazi S, Black JM, Abrams DA, Hoeft F, Menon V. Neurobiological underpinnings of math and reading learning disabilities. Journal of Learning Disabilities. 2013; 46: 549–569. doi: 10.1177/0022219413483174 23572008 10.1177/0022219413483174 PMC3795983

[19] Huang TH, Liu YC, Chang HC. Learning achievement in solving word-based mathematical questions through a computer-assisted learning system. Educational Technology & Society. 2012; 15: 248–259.

[20] Hecht SA, Torgesen JK, Wagner RK, Rashotte CA. The relations between phonological processing abilities and emerging individual differences in mathematical computation skills: A longitudinal study from second to fifth grades. Journal of Experimental Child Psychology. 2001; 79: 192–227. doi: 10.1006/jecp.2000.2586 11343408 10.1006/jecp.2000.2586

[21] Baddeley AD. Working memory. Psychology of Learning and Motivation. 1986; 8: 47–89.

[22] Anvari SH, Trainor LJ, Woodside J, Levy BA. Relations among musical skills, phonological processing, and early reading ability in preschool children. Journal of Experimental Child Psychology. 2002; 83: 111–130. 12408958 10.1016/s0022-0965(02)00124-8

[23] Passolunghi MC, Vercelloni B, Schadee H. The precursors of mathematics learning: Working memory, phonological ability and numerical competence. Cognitive Development. 2007; 22:165–184.

[24] Stevenson HW, Parker T, Wilkinson A, Hegion A, Fish E. Longitudinal study of individual differences in cognitive development and scholastic achievement. Journal of Educational Psychology. 1976; 68: 377.

[25] De Visscher A, Noël MP. Arithmetic facts storage deficit: The hypersensitivity to interference in memory hypothesis. Developmental Science. 2014; 17: 434–442. doi: 10.1111/desc.12135 24410798 10.1111/desc.12135

[26] McBride-Chang C, Bialystok E, Chong KK, Li Y. Levels of phonological awareness in three cultures. Journal of Experimental Child Psychology. 2004; 89: 93–111. doi: 10.1016/j.jecp.2004.05.001 15388300 10.1016/j.jecp.2004.05.001

[27] Purpura DJ, Ganley CM. Working memory and language: Skill-specific or domain-general relations to mathematics? Journal of Experimental Child Psychology. 2014; 122: 104–121. doi: 10.1016/j.jecp.2013.12.009 24549230 10.1016/j.jecp.2013.12.009

[28] Raman M. Coordinating informal and formal aspects of mathematics: Student behavior and textbook messages. The Journal of Mathematical Behavior. 2002; 21: 135–150.

[29] Song MJ, Ginsburg HP. The development of informal and formal mathematical thinking in Korean and US children. Child Development. 1987; 58: 1286–1296.

[30] Geary DC, Bailey DH, Hoard MK. Predicting mathematical achievement and mathematical learning disability with a simple screening tool the number sets test. Journal of Psychoeducational Assessment. 2009; 27: 265–279. doi: 10.1177/0734282908330592 20161145 10.1177/0734282908330592 PMC2731944

[31] Geary DC, Bailey DH, Littlefield A, Wood P, Hoard MK, Nugent L. First-grade predictors of mathematical learning disability: A latent class trajectory analysis. Cognitive development. 2009; 24: 411–429. 10.1016/j.cogdev.2009.10.001 PMC2813681 20046817

[32] Libertus ME, Feigenson L, Halberda J. Numerical approximation abilities correlate with and predict informal but not formal mathematics abilities. Journal of Experimental Child Psychology. 2013; 116: 829–838. doi: 10.1016/j.jecp.2013.08.003 24076381 10.1016/j.jecp.2013.08.003 PMC3796771

[33] Purpura DJ, Baroody AJ, Lonigan CJ. The transition from informal to formal mathematical knowledge: Mediation by numeral knowledge. Journal of Educational Psychology. 2013; 104: 53.

[34] Clements DH. Curriculum research: Toward a framework for “research-based curricula”. Journal for Research in Mathematics Education. 2007; 38: 35–70.

[35] LeFevre JA, Liu J. The role of experience in numerical skill: Multiplication performance in adults from Canada and China. Mathematical Cognition. 1997; 3: 31–62.

[36] Miller KF, Kelly M, Zhou X. Learning mathematics in China and the United States: Cross-cultural insights into the nature and course of mathematical development In: Handbook of mathematical cognition. New York: Psychology Press; 2005 pp. 163–178.

[37] Miller KF, Smith CM, Zhang H. Language and number: A longitudinal study of learning to count in Chinese and English. 2004; Forthcoming.

[38] Liu RD, Ding Y, Xu L, Wang J. Involvement of working memory in mental multiplication in Chinese elementary students. The Journal of Educational Research. 2017; 110: 1–11.

[39] LeFevre JA, Lei Q, Smith-Chant BL, Mullins DB. Multiplication by eye and by ear for Chinese-speaking and English-speaking adults. Canadian Journal of Experimental Psychology/Revue canadienne de psychologie expérimentale. 2001; 55: 277 11768852 10.1037/h0087374

[40] Keenan JM, Betjemann RS, Olson RK. Reading comprehension tests vary in the skills they assess: Differential dependence on decoding and oral comprehension. Scientific Studies of Reading. 2008; 12: 281–300.

[41] Wu X, Anderson RC, Li W, Wu X, Li H, Zhang J, et al Morphological awareness and Chinese children's literacy development: An intervention study. Scientific Studies of Reading. 2009; 13: 26–52.

[42] Cheung SK, McBride-Chang C. Evaluation of a parent training program for promoting Filipino young children’s number sense with number card games. Child Studies in Asia-Pacific Contexts. 2015; 5: 1–11.

[43] Chard DJ, Clarke B, Baker S, Otterstedt J, Braun D, Katz R. Using measures of number sense to screen for difficulties in mathematics: Preliminary findings. Assessment for Effective Intervention. 2005; 30: 3–14.

[44] Malofeeva E, Day J, Saco X, Young L, Ciancio D. Construction and evaluation of a number sense test with head start children. Journal of Educational Psychology. 2004; 96: 648–659.

[45] Bryant D, Bryant B, Gersten R, Scammacca N, Chavez M. Mathematics intervention for first-and second-grade students with mathematics difficulties: The effects of tier 2 intervention delivered as booster lessons. Remedial and Special Education. 2008; 29: 20–32.

[46] Clarke B, Shinn MR. A preliminary investigation into the identification and development of early mathematics curriculum based measurement. School Psychology Review. 2004; 33: 234–238.

[47] Ramani GB, Siegler RS. Promoting broad and stable improvements in low-income children’s numerical knowledge through playing number board games. Child Development. 2008; 79: 375–394. doi: 10.1111/j.1467-8624.2007.01131.x 18366429 10.1111/j.1467-8624.2007.01131.x

[48] Zhang J, McBride-Chang C, Tong X, Wong A, Shu H, Fong CYC. Reading with meaning: The contributions of meaning-related variables at the word and subword levels to early Chinese reading comprehension. Reading and Writing: An Interdisciplinary Journal. 2012; 25: 2183–2203.

[49] Raven JC, Court JH, Raven J, Kratzmeier H. Advanced progressive matrices: Raven-matrizen-test. 1994

[50] Raudenbush SW, Bryk AS. Hierarchical linear models: Applications and data analysis methods. Newbury Park, CA: Sage; 2002.

[51] Krull JL, Mackinnon DP. Multilevel modeling of individual and group level mediated effects. Multivariate Behavioural Reaearch. 2001; 36: 249–277. 10.1207/S15327906MBR3602_06 26822111

[52] Wen Z, Ye B. Analyses of mediating effects: The development of methods and models. Advances in Psychological Science. 2014; 22: 731–745.

[53] Purpura DJ, Napoli AR. Early numeracy and literacy: Untangling the relation between specific components. Mathematical Thinking and Learning. 2015; 17: 197–218.

[54] Mark W, Dowker A. Linguistic influence on mathematical development is specific rather than pervasive: Revisiting the Chinese number advantage in Chinese and English children. Frontiers in Psychology. 2015; 6: 1–9. doi: 10.3389/fpsyg.2015.00001 25767456 10.3389/fpsyg.2015.00203 PMC4341514

[55] Jordan NC, Kaplan D, Locuniak MN, Ramineni C. Predicting first-grade math achievement from developmental number sense trajectories. Learning Disabilities Research & Practice. 2007; 22: 36–46.

[56] LeFevre J, Fast L, Skwarchuk S, Smith-Chant BL, Bisanz J, Kamawar D, et al Pathways to mathematics: Longitudinal predictors of performance. Child Development. 2010; 81: 1753–1767. doi: 10.1111/j.1467-8624.2010.01508.x 21077862 10.1111/j.1467-8624.2010.01508.x

[57] Kaiser AP, Hancock TB. Teaching parents new skills to support their young children's development. Infants & Young Children. 2003; 16: 9–21.

[58] Starkey P, Klein A, Wakeley A. Enhancing young children’s mathematical knowledge through a pre-kindergarten mathematics intervention. Early Childhood Research Quarterly. 2004; 19: 99–120.

[59] Claessens A, Duncan G, Engel M. Kindergarten skills and fifth-grade achievement: Evidence from the ECLS-K. Economics of Education Review. 2009; 28: 415–427.

[60] Klein A, Starkey P, Clements D, Sarama J, Iyer R. Effects of a pre-kindergarten mathematics intervention: a randomized experiment. Journal of Research on Educational Effectiveness. 2008; 1: 155–178.

[61] Vandermaas-Peeler M, Ferretti L, Loving S. Playing the Ladybug Game: Parent guidance of young children's numeracy activities. Early Child Development and Care. 2012; 182: 1289–1307.

[62] Starkey P, Klein A. Fostering parental support for children's mathematical development: An intervention with Head Start families. Early Education and Development. 2000; 11:659–680.

[63] Purpura DJ, Lonigan CJ. Informal numeracy skills: The structure and relations among numbering, relations, and arithmetic operations in preschool. American Educational Research Journal. 2013; 50: 178–209.

[64] Tong X, McBride-Chang C, Wong AMY, Shu H, Reitsma P, Rispens J. Longitudinal predictors of very early Chinese literacy acquisition. Journal of Research in Reading. 2011; 34: 315–332.

